# A Phase II Study of Rucaparib Monotherapy in Nonmetastatic, Hormone-Sensitive Prostate Cancer Demonstrating “BRCAness” Genotype (ROAR)

**DOI:** 10.1093/oncolo/oyae030

**Published:** 2024-03-07

**Authors:** Kamal Kant Sahu, Haoran Li, Vinay Mathew Thomas, Mallory Benson, Ken Boucher, Sumati Gupta, Manish Kohli, Umang Swami, Neeraj Agarwal, Benjamin L Maughan

**Affiliations:** Division of Medical Oncology, Department of Internal Medicine, Huntsman Cancer Institute, University of Utah, Salt Lake City, UT, USA; Division of Medical Oncology, Department of Internal Medicine, University of Kansas Cancer Center, Westwood, KS, USA; Division of Medical Oncology, Department of Internal Medicine, Huntsman Cancer Institute, University of Utah, Salt Lake City, UT, USA; Division of Medical Oncology, Department of Internal Medicine, Huntsman Cancer Institute, University of Utah, Salt Lake City, UT, USA; Department of Internal Medicine, Huntsman Cancer Institute, University of Utah, Salt Lake City, UT, USA; Division of Medical Oncology, Department of Internal Medicine, Huntsman Cancer Institute, University of Utah, Salt Lake City, UT, USA; Division of Medical Oncology, Department of Internal Medicine, Huntsman Cancer Institute, University of Utah, Salt Lake City, UT, USA; Division of Medical Oncology, Department of Internal Medicine, Huntsman Cancer Institute, University of Utah, Salt Lake City, UT, USA; Division of Medical Oncology, Department of Internal Medicine, Huntsman Cancer Institute, University of Utah, Salt Lake City, UT, USA; Division of Medical Oncology, Department of Internal Medicine, Huntsman Cancer Institute, University of Utah, Salt Lake City, UT, USA

**Keywords:** prostate cancer, rucaparib, BRCAness, PARP

## Abstract

**Background:**

Both germline and somatic BReast CAncer gene (*BRCA*) mutations are poor prognostic markers in men with localized or metastatic prostate cancer. For instance, men with these mutations often are diagnosed with prostate cancer earlier and develop metastatic disease earlier compared with those who do not harbor similar mutations. Patients with germline alterations typically have more advanced disease and shorter overall survival (Castro E, Goh C, Olmos D, et al. Germline BRCA mutations are associated with higher risk of nodal involvement, distant metastasis, and poor survival outcomes in prostate cancer. *J Clin Oncol*. 2013;31(14):1748-1757. doi:10.1200/JCO.2012.43.1882). The risk of disease progression to metastatic disease is significant in patients with this genotype of prostate cancer. The percentage of patients free from metastatic disease was 90%, 72%, and 50%, respectively, compared with 97%, 94%, and 84% at 3, 5, and 10 years for patients with intact DNA repair (*P* < .001) (Castro E, Goh C, Leongamornlert D, et al. Effect of BRCA mutations on metastatic relapse and cause-specific survival after radical treatment for localised prostate cancer. *Eur Urol*. 2015;68(2):186-193. doi: 10.1016/j.eururo.2014.10.022). DNA damage repair non-*BRCA* mutations include alterations in genes such as *ATM, CHEK2, PALB2,* and *RAD51.* While less common than *BRCA* mutations, they have emerged as significant prognostic markers in prostate cancer. These *BRCA*ness mutations are associated with a higher risk of aggressive disease and poorer survival outcomes. Given the debilitating physical and psychological side effects of androgen deprivation therapy (ADT) in relatively younger men with prostate cancer, delaying ADT in these men may be an attractive strategy. Given the proven efficacy of polyadenosine diphosphate-ribose polymerase (PARP) inhibitors in the castration-resistant prostate cancersetting, PARP inhibitor monotherapy in a nonmetastatic castration-sensitive (nmCSPC) setting has the potential to delay metastasis and delay the onset of ADT related symptoms.

**Methods:**

This is a single-arm, single-center, open-label, phase II trial to assess the efficacy of rucaparib in patients with high-risk biochemically recurrent (BCR) nmHSPC, which was defined as PSA doubling time of <9 months, demonstrating a “BRCAness” genotype (*BRCA1/2* and other homologous recombination repair mutations). A total of 15 patients were intended to be enrolled, with an expected enrollment duration of 12 months. Patients were given rucaparib 600 mg orally twice daily and were allowed to remain on study treatment until PSA progression defined by Prostate Cancer Working Group 3, with 2 years of follow-up after study treatment. We anticipated a total of 2-3 years until completion of the clinical trial. The primary endpoint was to assess the PSA progression-free survival (PSA-PFS). The secondary endpoints of the study were safety, the proportion of patients with a PSA 50% response (PSA 50), and an undetectable PSA. A 4-week treatment duration comprised one cycle.

**Results:**

The study started enrolling in June 2019 and was prematurely terminated in June 2022 after the accrual of 7 patients because of changing standard of care treatments with the introduction of next-generation scans, eg, prostate-specific membrane antigen positron emission tomography (PSMA-PET). Seven patients were enrolled in the study with the following pathogenic alterations: *ATM* (*n* = 3), *BRCA2* (*n* = 2), *BRCA1* (*n* = 1), *BRIP1* (*n* = 1), and *RAD51* (*n* = 1). The median duration of follow-up was 18 months. A median of 20 cycles (range 4-42) was completed, median PSA-PFS was 35.37 months (95% CI, 0-85.11 months). In total, 2 patients achieved PSA50; both also achieved nadir PSA as undetectable. Grade ≥ 3 adverse events (AEs) were anemia and rash (in 1 patient each). No dose-limiting toxicities or severe AEs were seen.

**Conclusion:**

Rucaparib demonstrated acceptable toxicity and efficacy signal as an ADT-sparing approach in patients with biochemically recurrent nonmetastatic prostate cancer. It is currently challenging to understand the optimal value of systemic therapy in this disease setting due to the rapidly changing standard of care. Additionally, there are relatively few patients with BRCAness who present with nonmetastatic hormone-sensitive prostate cancer (ClinicalTrials.gov Identifier: NCT03533946).

## Teaser

This article reports clinical trial results of a phase II trial assessing the efficacy of rucaparib in patients with high-risk biochemically recurrent nonmetastatic hormone-sensitive prostate cancer.

**Lessons Learned**

- Rucaparib demonstrated acceptable toxicity and efficacy signal in biomarker selected patients with prostate cancer with biochemical recurrence.
- No treatment-related severe adverse events were seen with rucaparib monotherapy in men with systemic treatment naïve high-risk non-metastatic castration sensitive prostate cancer.

## Discussion

Polyadenosine diphosphate-ribose polymerase inhibitor (PARPi) therapy has demonstrated significant clinical activity in patients with metastatic castration-resistant prostate cancer (mCRPC) which harbors DNA repair deficiencies [termed DDR deficient or BRCAness].^1–3^ This has prompted additional studies incorporating PARPi earlier in the disease course (ie, in a castration-sensitive setting). For instance, TALAPRO-3 is a phase III clinical trial of enzalutamide plus talazoparib in patients with DDR gene mutated metastatic castration-sensitive prostate cancer (mCSPC).^4^ Currently, treatment for patients with biochemical recurrence (nonmetastatic castration-sensitive prostate cancer) is not specifically tailored differently for patients with BRCAness versus those without BRCAness. Standard of care includes treating all patients with androgen deprivation therapy (ADT) or combined with enzalutamide. Hence, there is an unmet need to tailor prostate cancer therapy based on BRCAness.

ADT contributes to the quality of life decrements in patients with prostate cancer.^5^ It leads to more fatigue and frailty compared with drugs used for treatment intensification with novel hormonal therapies (NHTs: abiraterone, apalutamide, or enzalutamide).^6–10^ In contrast, PARPi has less impact on quality of life than hormone therapy and is generally a very well-tolerated intervention.^11^

We conducted this study with the aim to intervene early with PARPi in patients with BCR non-metastatic CSPC with BRCAness-related alterations to both achieve disease control while concurrently avoiding the toxicity of ADT treatment. All patients were treated with rucaparib monotherapy, and none were treated with ADT. The primary endpoint was to assess the PSA progression-free survival (PSA-PFS).

This trial demonstrated a signal of activity in biomarker-selected patients with rucaparib monotherapy while maintaining a good safety profile. The best responders were patients with BRCA 1/2 and RAD 51 mutations as measured by prolonged PSA-PFS. No robust clinical activity was seen in patients with only ATM mutations. PSA 50% response rates were observed in those patients with durable PSA responses. The study was prematurely terminated after the enrollment of seven patients when PSMA-PET/CT imaging was approved by the Food and Drug Administration for patients with elevated serum PSA levels following definitive treatment, which changed the standard of care for these patients. Currently, adjuvant/salvage radiation therapy, palliative ADT, or surveillance remains the standard of care in patients with PSA recurrence following definitive treatment for localized prostate cancer. These hypothesis-generating data suggest that rucaparib may be an alternative option in this selected group of patients with high-risk biochemically recurrent CSPC, with specific BRCAness genotypes who prefer to delay ADT.

**Table:** 

Trial Information	
Disease	Prostate Cancer
Stage of disease/treatment	Nonmetastatic, castration sensitive with biochemical recurrence/rucaparib monotherapy
Prior therapy	None
Type of study	Single-arm, single-center, open label, phase II trial
Primary endpoint	To assess PSA progression-free survival (PSA-PFS)
Secondary endpoints	To assess the safety of rucaparib in patients with biochemically recurrent non-metastatic castration-sensitive prostate cancer. To assess the proportion of patients with a 50% reduction in PSA levels (PSA50) compared to the baseline value at the time of study nrolment. To assess the proportion of patients with an undetectable PSA after initiation of PARPi therapy at 6 and 12 months. To evaluate overall survival.

## Additional Details of Endpoints or Study Design

### Patients

**Figure 1.**
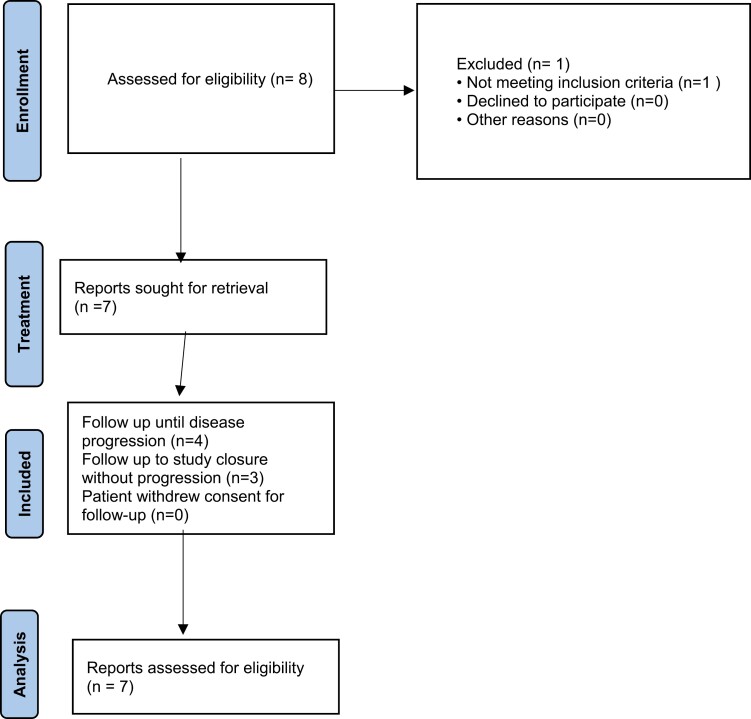
Patient flowchart.

This was a phase II study in high-risk BCR non-metastatic CSPC setting with BRCAness genotype to investigate the efficacy of rucaparib monotherapy. High-risk biochemically recurrence was defined as PSA doubling time of <9 months. Target was to enroll 15 evaluable patients with BRCAness. Detailed inclusion and exclusion criteria can be found in Fig. 1.

### Inclusion Criteria

- Castration-sensitive, histologically proven adenocarcinoma of the prostate with BRCAness (defined as an alteration in one or more of the following genes BARD1, BRCA1, BRCA2, BRIP1, CHEK1, CHEK2, FANCA, NBN, PALB2, RAD51C, RAD51D, RAD51, RAD51B) from soft-tissue based genomic testing or liquid biopsy-based genomic or genetic testing. Pathogenic or likely pathogenic alterations were accepted.
- ECOG/Zubrod score of 0-2.
- At a minimum, patients must have received definitive local therapy with curative intent (ie, prostatectomy and/or radiation therapy) with or without concurrent systemic therapy with ADT.
- Testosterone level of >50 ng/dL.
- Age of patient of at least 18 years old at the time the informed consent form was signed.
- Demonstrate adequate organ function, with all screening labs performed within 28 days of treatment initiation.
- Rising PSA without radiographic evidence of metastatic disease and with PSA doubling time of ≤10 months. PSA progression was confirmed based on at least 2-time points taken at least one week apart to confirm the rising trend.
- Recovery to baseline or Grade ≤1 Common Terminology Criteria for Adverse Events (CTCAE v5.0) from toxicities related to any prior treatments within the context of their definitive local therapy for their prostate cancer unless AE(s) is clinically nonsignificant and/or stable on supportive therapy.

### Exclusion Criteria

- Patients with metastases defined by conventional scans (Computed Tomography Scan, Magnetic resonance imaging, nuclear medicine bone scan). Diseases identified on molecular imaging (eg, fluciclovine-PET) were not considered exclusionary.
- Arterial or venous thrombi (including a cerebrovascular accident), myocardial infarction, admission for unstable angina, cardiac angioplasty, or stenting within the last 90 days prior to screening.
- Pre-existing duodenal stent, recent (within ≤3 months) or existing bowel obstruction, and/or any gastrointestinal disorder or defect that would, in the opinion of the investigator, interfere with absorption of rucaparib.
- Inability to swallow tablets.
- Evidence or history of clinically significant bleeding disorder per the determination of the treating investigator.
- Prior systemic therapy within the past 30 days prior to day 1 (or 5 half-lives of the drug, whichever is shorter).
- Diagnosis of another malignancy within 2 years before the first dose of study treatment only if cancer will either interfere with patient safety or interfere with the primary endpoint, per the judgment of the principal investigator. Patients who have been diagnosed with superficial skin cancers, or localized, low-grade tumors deemed cured or with a prolonged natural history (eg, estimated overall survival >5 years), were included.
- Prior treatment with any PARPi, mitoxantrone, cyclophosphamide, or any platinum-based chemotherapy.

### Trial Design and Treatment

This is a single-arm, single-center, open-label, phase II trial to assess the efficacy of rucaparib in high-risk non-metastatic CSPC with “BRCAness” genotype. Treatment with rucaparib was given on cycle 1 day 1 and continued at 600 mg per oral twice daily. No patients were allowed to be on ADT therapy either by surgical castration or with GnRH agonist or antagonist or on androgen synthesis blocker or androgen receptor antagonists during the study period.

Duration of administration: Patients were allowed to remain on study treatment until unacceptable toxicity, PSA progressive disease (unless they meet the criteria for continued treatment post-progression), or patient withdrawal.

Dose modifications and safety assessments: adjustment of rucaparib dosage was permissible for adverse event (AE) management, and study specific protocol guidelines for dose modifications were followed. Delays in dosage administration were sanctioned for both therapeutic agents to effectively address adverse events (AEs). AEs and laboratory abnormalities were systematically categorized and evaluated based on the grading system delineated in the National Cancer Institute (NCI) CTCAE v5.0. Dose-limiting toxicity (DLT) was characterized by any adverse event of severity grade ≥3 manifesting within the initial 3 weeks of treatment, and its association with either of the experimental compounds was to be determined by the principal investigator. Interruption of rucaparib treatment was permissible under the following circumstances, prompting consideration or implementation of a dosage reduction: (1) hematologic toxicity of grade 3 or 4 and (2) non-hematologic toxicity of grade 3 or 4.

Patients received treatment until the point of disease progression, which was defined as the emergence of new metastatic instances on conventional imaging or a confirmed rise in prostate-specific antigen (PSA) levels. Alternatively, treatment was halted in the presence of unacceptable toxicity or upon meeting other stipulated withdrawal criteria outlined in the study protocol. Discontinuation of treatment was mandated for adverse events (AEs) categorized as grade 4, except for isolated laboratory values that deviated from the norm and were determined to be unrelated to the administered treatment. Such deviations lacked clinical correlation and were resolved within a span of 7 days under medical supervision.

Furthermore, patients were allowed to continue receiving treatment beyond the point of disease progression if the treating investigator perceived potential clinical benefit. The scope of safety assessments encompassed the meticulous documentation of AEs, comprehensive physical examinations, and a battery of clinical laboratory tests including hematology analyses, hepatic panels, and serum chemistries. Additionally, concurrent medication usage was duly recorded for each patient. Within this context, a serious adverse event (SAE) was precisely defined as any unfavorable incident leading to fatal consequences, a life-threatening situation, necessitating inpatient hospitalization, extending an ongoing hospital stay, resulting in enduring or significant impairment, contributing to a congenital anomaly or birth defect, or otherwise deemed medically imperative.

### Outcomes

For the outcome assessment, patients must have received at least one full cycle of rucaparib and have PSA assessed after that cycle to be evaluable for efficacy. PSA levels were monitored monthly PSA progression-free survival (PSA-PFS) was defined as the time from study enrollment until the time of PSA progression as defined by PCWG3 criteria or death.^12^ Patients who started another anti-cancer treatment prior to PSA progression or who completed one year of follow-up without PSA progression had their data censored at that time point. The primary objective of this phase II study was to assess PSA progression-free survival (PSA-PFS) upon receipt of rucaparib monotherapy in BCR non-metastatic CSPC setting with BRCAness genotype. The secondary objective was to assess the safety of rucaparib, proportion of patients with a 50% reduction in PSA levels from baseline, proportion of patients with an undetectable PSA after initiation of PARPi therapy at 6 and 12 months, and to evaluate overall survival (OS).

### Statistical Analysis

#### Primary Objectives

Patient demographics and baseline characteristics were described using frequencies and percentages (%) for categorical variables and medians and interquartile ranges for continuous variables. The total sample size was planned to enroll 15 patients. The null hypothesis was that the median PSA-PFS would be 3 months. With an alternative hypothesis that the median PSA-PFS would be 12 months, a sample size of 15 provides 90% power at one-sided alpha = 0.05.

#### Secondary Objectives

Descriptive analysis methods were used to analyze tabulated safety characteristics. Kaplan-Meier methods and associated confidence intervals were used to analyze PSA, PFS, and OS. The proportion of subjects who achieve PSA50 are reported along with a 95% confidence interval. The proportion of subjects with an undetectable PSA after initiation of PARP therapy at 6 and 12 months are assessed using Kaplan-Meier methods and associated confidence intervals. The proportion of subjects who exhibited an objective response was reported along with an exact binomial confidence interval.

**Table:** 

Drug Information	
Generic/working name	Rucaparib
Company name	Clovis Oncology, Inc.
Drug type	Inhibitor of poly (adenosine diphosphate [ADP]-ribose) polymerase (PARP)
Drug class	Small molecule
Dose	600 mg twice daily, oral
Schedule of administration	28 days treatment per cycle

**Table:** 

Patient Characteristics	
Number of patients, male	7
Number of patients, female	0
Stage	Localized/non-metastatic with biochemical recurrence in castration sensitive setting
Age: median (range)	69 (59-75) years
Number of prior systemic therapies	0
Performance status: ECOG	0: 5 1: 2 3: 0 4: 0
Cancer types or histologic subtypes	Histologically proven adenocarcinoma of the prostate, 7 (100%)

### Notes

The baseline demographics and clinical characteristics are summarized above. The median age of patients in our cohort was 69 years (59-75 years). The median time from the initial diagnosis of local disease to rucaparib treatment was 45.23 months (30.03- 249.93 months). The median PSA at initial diagnosis, and at the time of study enrollment was 9.43 ng/mL (2.3-53.77 ng/mL), and 0.9 ng/ml (0.07-9.2 ng/mL), respectively. Regarding the previous treatment, all patients underwent prostatectomy and adjuvant/salvage radiation. Prior androgen deprivation therapy exposure was noted in 4 patients given concurrently with radiation (Table 1). Molecular testing results are as follows: ATM (*n* = 3), BRCA2 (*n* = 2), BRCA1 (*n* = 1), BRIP1 (*n* = 1), RAD51 (*n* = 1). One patient had both ATM and RAD51 mutations. All these mutations are germline except for the BRIP 1 which was somatic.

**Table 1.** Demographics, disease characteristics, and treatment details before enrollment in the study.

Patient	Age on study (years)	Race	PSA at baseline (ng/ml)	PSA at progression or censor(ng/ml)	Gleason score	Specific gene mutation(s)	Previous prostatectomy	Previous radiation therapy	Previous androgen deprivation therapy
1	75	White	7	11	7 (3 + 4)	BRIP1	Yes	Yes	No
2	69	White	0.9	1.9	6 (3 + 3)	BRCA2	Yes	Yes	Yes
3	60	White	9.2	11.9	7 (4 + 3)	ATM	Yes	Yes	No
4	71	White	0.3	2.3	7 (3 + 4)	BRCA1	Yes	Yes	Yes
5	64	White	4	7.2	9 (5 + 4)	ATM	Yes	Yes	Yes
6	70	White	0.2	0	8 (4 + 4)	ATM; RAD51	Yes	Yes	Yes
7	59	White	0.07	0	8 (4 + 4)	BRCA2	Yes	Yes	No

**Table:** 

Primary Assessment Method	
Title	PSA progression-free survival (PSA-PFS)
Number of patients screened	8
Number of patients enrolled	7
Number of patients evaluable for toxicity	7
Number of patients evaluated for efficacy	7
Evaluation method	PSA level
PSA-PFS	35.37 months (95% CI, 0-85.11 months)
Response duration	In 2 patients who achieved PSA 50%, the median response duration was 16.83 months (range: 16.87-17.80 months).
Duration of treatment	17.5 months

### Outcome Notes

**Figure 2.**
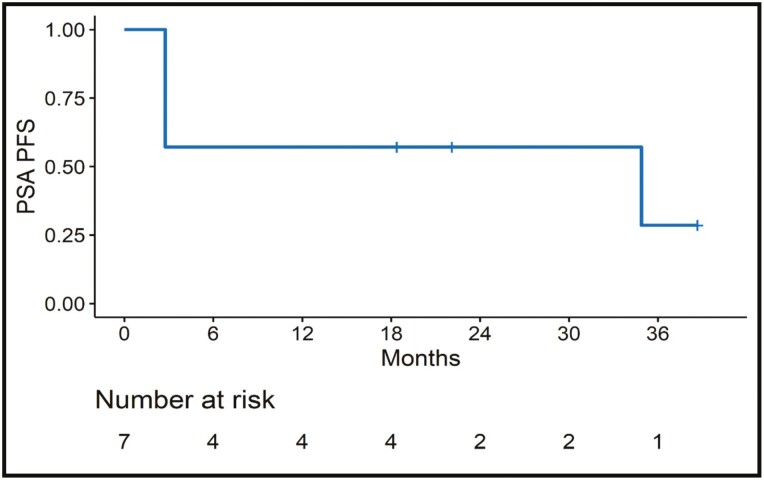
PSA progression-free survival (Kaplan-Meier survival analysis).

**Figure 3.**
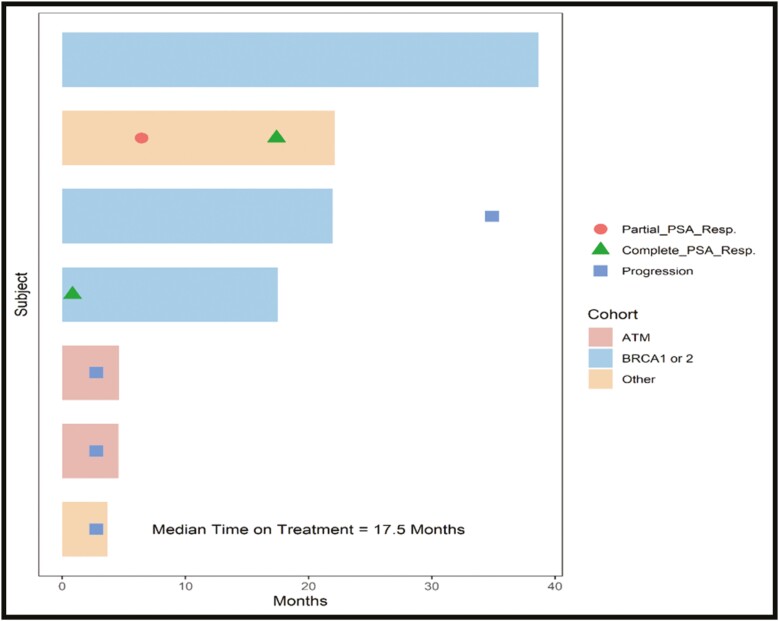
Duration of response of the patients (Swimmer’s plot).

**Figure 4.**
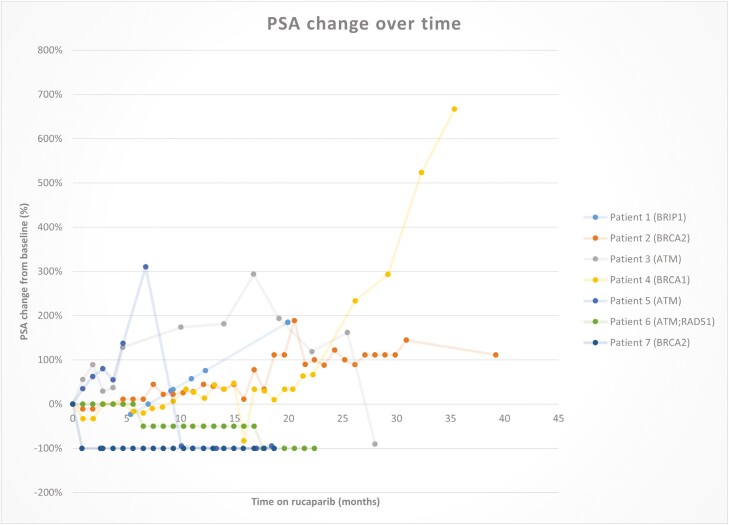
PSA change over time.

The median duration of follow-up was 18 months. A median of 20 cycles (range 4-42) was completed, median PSA-PFS was 35.37 months (95% CI, 0-85.11 months) (Figure 2). At the time of data censoring, there was no PSA progression in 3 patients (2 patients had BRCA2, and 1 had ATM/RAD 51). Two patients achieved the PSA 50% (BRCA 2, and ATM/RAD 51), with both patients achieving an undetectable PSA level during the treatment period (Table 2 and Figure 3). The rate of PSA change over time during follow-up has been described in Figure 4.

**Table 2.** Treatment duration with rucaparib, and PSA responses.

Patient	Specific gene mutation(s)	Cycles of rucaparib completed	PSA progression Y/N	50% PSA reduction achieved Y/N	Undetectable PSA at 6m Y/N	Undetectable PSA at 12m Y/N	Undetectable PSA during treatment Y/N
1	*BRIP1*	4	Yes	No	No	No	No
2	*BRCA2*	42	No	No	No	No	No
3	*ATM*	5	Yes	No	No	No	No
4	*BRCA1*	24	Yes	No	No	No	No
5	*ATM*	5	Yes	No	No	No	No
6	*ATM; RAD51*	24	No	Yes	No	No	Yes
7	*BRCA2*	20	No	Yes	Yes	Yes	Yes

**Table:** 

Secondary Assessment Method	
Title	Safety of rucaparib
Number of patients screened	8
Number of patients enrolled	7
Number of patients evaluable for toxicity	7
Number of patients evaluated for efficacy	7
Evaluation method	Monitoring and recording all adverse events and serious adverse events using the CTCAE version 5.0
Safety assessment	Dose-limiting toxicity: 0/7 Severe adverse events: 0/7 All grade adverse events: 7/7 Grade ≥ 3 adverse events: 2/7 (28.57%)

### Outcome Notes

Adverse events (AEs): All patients developed adverse events (AEs), mostly grade 1-2 AEs. The most common grade 1 AE was fatigue (5/7), followed by dysgeusia (4/7). Two patients developed grade 3 AEs (anemia and acneiform rash each) [Table 3].
Drug interruptions: There were no drug interruptions in any patient.

**Table 3.** Adverse events per Common Terminology Criteria for Adverse Events (CTCAE) 5.0.

CTCAE Term (attributed to rucaparib)	G1-2 *n* (%)	G3 *n* (%)	All grades *n* (%)
Alanine aminotransferase increased	2 (28.6)		2 (28.6)
Alkaline phosphatase increased	1 (14.3)		1 (14.3)
Anemia	1 (14.3)	1 (14.3)	2 (28.6)
Anorexia	1 (14.3)		1 (14.3)
Aspartate aminotransferase increased	1 (14.3)		1 (14.3)
Dry mouth	1 (14.3)		1 (14.3)
Dysgeusia	4 (57.1)		4 (57.1)
Fatigue	5 (71.4)		5 (71.4)
Mucositis oral	1 (14.3)		1 (14.3)
Nausea	3 (42.9)		3 (42.9)
Photosensitivity	1 (14.3)		1 (14.3)
Rash acneiform	1 (14.3)	1 (14.3)	1 (14.3)
Skin and subcutaneous tissue disorders—Other (blistering hands, int)	1 (14.3)		1 (14.3)
White blood cells decreased	2 (28.6)		2 (28.6)
Total patients affected	7 (100)	2 (28.6)	7 (100)

## Assessment, Analysis, and Discussion

**Table:** 

Completion	Study terminated prior to completion
Investigator’s assessment	Active but results overtaken by other developments

Prostate cancer stands as one of the most prevalent malignancies among men. In 2023, there will be an estimated 288 300 new cases resulting in 34 700 fatalities in the United States.^13^ While early-stage prostate cancer can often be treated with local therapy, such as surgery or radiation therapy, advanced or metastatic prostate cancer requires systemic therapy.^14^Patients with germline alterations typically have more advanced disease and shorter overall survival.^15^ The risk of disease progression to metastatic disease is significant in patients with this genotype of prostate cancer. The percentage of patients free from metastatic disease was 90%, 72%, and 50%, respectively, compared with 97%, 94%, and 84% at 3, 5, and 10 years for patients with intact DNA repair (P < .001).^16^ Recent advancements have showcased significant clinical efficacy in managing metastatic prostate cancer by targeting the PARP pathway.

Our trial evaluated the efficacy and safety of rucaparib monotherapy for patients diagnosed with high-risk, nmCSPC, and exhibiting the BRCAness genotype. For this study, eligible mutations included were alterations in one or more of the following genes: BARD1, BRCA1, BRCA2, BRIP1, CHEK1, CHEK2, FANCA, NBN, PALB2, RAD51C, RAD51D, or RAD51, RAD51B from soft tissue based genomic testing or liquid biopsy-based genomic or genetic testing. Also, pathogenic, or likely pathogenic alterations were accepted. The study enrolled a cohort of 7 patients with the primary objective of PSA progression-free survival (PSA-PFS). Secondary endpoints included were safety assessment defined per CTCAE (version 5.0), the proportion of patients experiencing a 50% decrease in PSA (PSA50), and the attainment of an undetectable PSA at any time after starting rucaparib.

We hypothesized that PARPi monotherapy in patients with nmCSPC will yield acceptable disease control, as reflected by PSA response. Notably, 3 patients demonstrated sustained PSA response, including 2 individuals with BRCA2 mutations and 1 harboring ATM/RAD 51 mutations. Remarkably, no severe adverse events (SAEs) were observed. The adverse event profile of rucaparib remained consistent with the previous studies and no new safety signals were identified.^17^ The rate of treatment-related adverse events (TRAEs) of moderate grade was acceptable, with no instances of grade 4-5 TRAEs reported. The promising outcomes observed in patients with BRCA2 and RAD 51 mutations suggest the potential benefits of rucaparib in this subgroup of patients.

Another study that also investigated PARPi (Olaparib) monotherapy in the setting of BCR prostate cancer (s/p prostatectomy) was reported recently by Marshall et al.^18^ This phase 2 trial investigated olaparib therapy in men with high-risk BCR prostate cancer post-prostatectomy. The trial enrolled 51 patients irrespective of their DDR mutation status. The tissue-based sequencing identified 27/51 patients with DDR mutations, predominantly involving BRCA2, CHEK2, and ATM genes. The overall PSA50 response rate was 24%, with a substantial 44% response rate in the DDR [+] group and no responses in the DDR [−] group. Notably, all patients with BRCA2 mutations (*n* = 11) achieved a PSA50 response. Furthermore, the DDR [+] group exhibited a longer median PSA progression-free survival (22 months) compared to the DDR [−] group (13 months). The study concluded that olaparib is a safe and effective treatment for men with BCR prostate cancer and DDR mutations, specifically highlighting its efficacy in patients with BRCA2 mutations, thereby encouraging further research into this treatment option. The total number of patients included in both studies is low. However, the results in the biomarker-selected groups are entirely consistent suggesting that PARPi is effective as monotherapy.

With the evolution of new advanced treatments for metastatic prostate cancer, patient survival rates have witnessed significant improvement. Median overall survival (OS) has extended from a minimum of 5 years for those diagnosed with metastatic disease to over 8 years for individuals progressing to metastatic status following definitive local interventions.^19,20^ The long-term repercussions of sustained ADT are progressively becoming evident and of clinical significance in the context of enhanced disease control, and prolonged survival. ADT escalates the susceptibility to cardiovascular disease, osteoporosis, and metabolic syndrome.^21^ Identifying therapeutic strategies that can effectively control prostate cancer and delay ADT use can further improve the care of patients with prostate cancer by both directly controlling metastatic disease progression, while simultaneously circumventing the health complications entailed by prolonged ADT. Our study represents a pioneering effort to uncover ADT-sparing options for patients afflicted with high-risk BCR prostate cancer.

Our findings correlate with larger prospective trials of PARP inhibitors conducted in prostate cancer in metastatic setting. Notably, the PROfound study, a phase III randomized trial, demonstrates the remarkable efficacy of olaparib. The median radiographic progression-free survival (rPFS) was 7.4 months compared to 3.6 months with and without PARPi, respectively.^2^ Similar outcomes were observed in the TRITON study in patients specifically harboring BRCA1/2 mutations.^1^ More recently, the TALAPRO-2 trial further reaffirms the efficacy of PARPi treatment (talazoparib plus enzalutamide) in these patients. The median rPFS was not reached with talazoparib and enzalutamide combination as compared to 21.9 months for placebo plus enzalutamide. In patients, whose tumors had BRCA gene alterations, integration of talazoparib with enzalutamide yielded a notable 77% lower risk of radiographic progression or death when compared with those in the placebo group.^22^These investigations underscore the clinical benefit of PARP inhibitors in selected patients with DNA damage repair gene mutations.

Inclusion of ADT remains the care standard for patients with advanced or metastatic prostate cancer. However, its implementation comes with an array of adverse effects including fatigue, diminished libido, cardiovascular events, and osteoporosis.^23^ The outcomes from the ROAR study suggest that PARPi therapy might serve as a novel approach for patients to postpone ADT initiation while concurrently providing durable disease control. This might enhance patients’ quality of life and delay the onset of ADT-linked adverse effects.

The variability in response observed among patients could be attributed to specific gene mutations. In our study, the best responses were observed in patients with BRCA1/2 and RAD51 mutations. In contrast, patients with exclusively ATM mutations did not exhibit robust clinical activity. This finding was consistent with existing literature, particularly emphasizing the discordant response rates associated with distinct mutations.^22^ Patients harboring BRCA1/2 mutations tend to exhibit more frequent and enduring responses to therapy, setting them apart from those with other mutations. It underscores the significance of understanding the genetic makeup of an individual’s tumor when predicting therapeutic outcomes.

It is important to acknowledge the limitations inherent in our study, including the very limited sample size and absence of a comparative arm making it impossible to draw any definitive conclusions. Furthermore, the study’s premature termination further limits the analysis of duration of response, which is important for patients early in the disease course given the favorable long-term prognosis. Further studies with larger sample sizes and randomized controlled designs are imperative to corroborate both the safety and efficacy of rucaparib monotherapy within this patient subset. Additionally, it is noteworthy that the study solely examined a confined spectrum of BRCAness gene alterations, leaving the response to other DDR gene mutations unknown.

The potential next steps from this manuscript involve several key areas. First, with the advent of newer diagnostic technologies like PSMA PET/CT, future studies can more accurately identify and stratify patients with nonmetastatic castration-sensitive prostate cancer (nmCSPC). This advanced imaging technology could aid in better selecting patients who might benefit most from PARP inhibitor therapy, particularly those with “BRCAness” genotypes. Second, given the small cohort in our current study, larger-scale trials are needed to robustly investigate the differential responses to PARP inhibitors in patients with monoallelic versus biallelic BRCA 1/2 mutations. Such differentiation could provide deeper insights into the biological mechanisms driving treatment efficacy. Finally, the recent approval of enzalutamide in this setting opens new possibilities for combining PARPi with NHTs. This combination could potentially enhance therapeutic efficacy, targeting different pathways involved in prostate cancer progression.

## Conclusion

In view of the profound physiological and psychological side effects of ADT in men with BRCAness prostate cancer, delaying ADT may be an attractive therapeutic strategy. Despite the small sample size, these results are consistent with other published studies of PARPi in prostate cancer. The use of PARPi monotherapy presents an opportunity for exploration in future clinical trials. The ROAR study adds to the growing body of evidence supporting the use of PARP inhibitors in patients with prostate cancer and BRCAness.

## Data Availability

The data underlying this article will be shared on reasonable request to the corresponding author.

## References

[1] Fizazi K , Piulats JM, Reaume MN, et al; TRITON3 Investigators. Rucaparib or physician’s choice in metastatic prostate cancer. N Engl J Med. 2023;388(8):719-732. 10.1056/NEJMoa2214676 36795891 PMC10064172

[2] Hussain M , Mateo J, Fizazi K, et al; PROfound Trial Investigators. Survival with olaparib in metastatic castration-resistant prostate cancer. N Engl J Med. 2020;383(24):2345-2357. 10.1056/NEJMoa2022485 32955174

[3] Mateo J , Carreira S, Sandhu S, et al. DNA-repair defects and olaparib in metastatic prostate cancer. N Engl J Med. 2015;373(18):1697-1708. 10.1056/NEJMoa1506859 26510020 PMC5228595

[4] Agarwal N , Azad A, Fizazi K, et al. Talapro-3: a phase 3, double-blind, randomized study of enzalutamide (ENZA) plus talazoparib (TALA) versus placebo plus enza in patients with DDR gene mutated metastatic castration-sensitive prostate cancer (mCSPC). J Clin Oncol. 2022;40(6_suppl):TPS221-TPSTPS.

[5] Cheung AS , de Rooy C, Hoermann R, et al. Quality of life decrements in men with prostate cancer undergoing androgen deprivation therapy. Clin Endocrinol (Oxf). 2017;86(3):388-394. 10.1111/cen.13249 27696495

[6] Stockler MR , Martin AJ, Davis ID, et al; ENZAMET Trial Investigators and the Australian and New Zealand Urogenital and Prostate Cancer Trials Group (ANZUP). Health-related quality of life in metastatic, hormone-sensitive prostate cancer: ENZAMET (ANZUP 1304), an international, randomized phase III trial led by ANZUP. J Clin Oncol. 2022;40(8):837-846. 10.1200/JCO.21.00941 34928708 PMC8906451

[7] Rush HL , Murphy L, Morgans AK, et al. Quality of life in men with prostate cancer randomly allocated to receive docetaxel or abiraterone in the STAMPEDE trial. J Clin Oncol. 2022;40(8):825-836. 10.1200/JCO.21.00728 34757812 PMC7612717

[8] Agarwal N , McQuarrie K, Bjartell A, et al. Apalutamide plus androgen deprivation therapy for metastatic castration-sensitive prostate cancer: analysis of pain and fatigue in the phase 3 TITAN study. J Urol. 2021;206(4):914-923. 10.1097/JU.0000000000001841 34039013

[9] Oudard S , Hadaschik B, Saad F, et al. Health-related quality of life at the SPARTAN final analysis of apalutamide for nonmetastatic castration-resistant prostate cancer patients receiving androgen deprivation therapy. Eur Urol Focus. 2022;8(4):958-967. 10.1016/j.euf.2021.08.005 34479838

[10] Smith MR , Shore N, Tammela TL, et al. Darolutamide and health-related quality of life in patients with non-metastatic castration-resistant prostate cancer: an analysis of the phase III ARAMIS trial. Eur J Cancer. 2021;154:138-146. 10.1016/j.ejca.2021.06.010 34273811

[11] Thiery-Vuillemin A , de Bono J, Hussain M, et al. Pain and health-related quality of life with olaparib versus physician’s choice of next-generation hormonal drug in patients with metastatic castration-resistant prostate cancer with homologous recombination repair gene alterations (PROfound): an open-label, randomised, phase 3 trial. Lancet Oncol. 2022;23(3):393-405. 10.1016/S1470-2045(22)00017-1 35157830

[12] Scher HI , Morris MJ, Stadler WM, et al; Prostate Cancer Clinical Trials Working Group 3. Trial design and objectives for castration-resistant prostate cancer: updated recommendations from the prostate cancer clinical trials working group 3. J Clin Oncol. 2016;34(12):1402-1418. 10.1200/JCO.2015.64.2702 26903579 PMC4872347

[13] Siegel RL , Miller KD, Wagle NS, Jemal A. Cancer statistics, 2023. CA Cancer J Clin. 2023;73(1):17-48. 10.3322/caac.21763 36633525

[14] McKay RR , Feng FY, Wang AY, Wallis CJD, Moses KA. Recent advances in the management of high-risk localized prostate cancer: local therapy, systemic therapy, and biomarkers to guide treatment decisions. Am Soc Clin Oncol Educ Book. 2020(40):e241-e252. 10.1200/edbk_279459 PMC10182417 32412803

[15] Castro E , Goh C, Olmos D, et al. Germline BRCA mutations are associated with higher risk of nodal involvement, distant metastasis, and poor survival outcomes in prostate cancer. J Clin Oncol. 2013;31(14):1748-1757. 10.1200/JCO.2012.43.1882 23569316 PMC3641696

[16] Castro E , Goh C, Leongamornlert D, et al. Effect of BRCA mutations on metastatic relapse and cause-specific survival after radical treatment for localised prostate cancer. Eur Urol. 2015;68(2):186-193. 10.1016/j.eururo.2014.10.022 25454609

[17] Coleman RL , Oza AM, Lorusso D, et al; ARIEL3 investigators. Rucaparib maintenance treatment for recurrent ovarian carcinoma after response to platinum therapy (ARIEL3): a randomised, double-blind, placebo-controlled, phase 3 trial. Lancet. 2017;390(10106):1949-1961. 10.1016/S0140-6736(17)32440-6 28916367 PMC5901715

[18] Marshall CH , Teply BA, Lim SJ, et al. Phase 2 study of olaparib (without ADT) in men with biochemically recurrent prostate cancer (BCR) after prostatectomy. J Clin Oncol. 2023;41(16_suppl):5087.

[19] Sweeney CJ , Chen YH, Carducci M, et al. Chemohormonal therapy in metastatic hormone-sensitive prostate cancer. N Engl J Med. 2015;373(8):737-746. 10.1056/NEJMoa1503747 26244877 PMC4562797

[20] Duchesne GM , Woo HH, Bassett JK, et al. Timing of androgen-deprivation therapy in patients with prostate cancer with a rising PSA (TROG 03.06 and VCOG PR 01-03 [TOAD]): a randomised, multicentre, non-blinded, phase 3 trial. Lancet Oncol. 2016;17(6):727-737. 10.1016/S1470-2045(16)00107-8 27155740

[21] Hershman DL , Unger JM, Wright JD, et al. Adverse health events following intermittent and continuous androgen deprivation in patients with metastatic prostate cancer. JAMA Oncol. 2016;2(4):453-461. 10.1001/jamaoncol.2015.4655 26720308 PMC4852142

[22] Agarwal N , Azad AA, Carles J, et al. Talazoparib plus enzalutamide in men with first-line metastatic castration-resistant prostate cancer (TALAPRO-2): a randomised, placebo-controlled, phase 3 trial. Lancet. 2023;402(10398):291-303. 10.1016/S0140-6736(23)01055-3 37285865

[23] Bolla M , Collette L, Blank L, et al. Long-term results with immediate androgen suppression and external irradiation in patients with locally advanced prostate cancer (an EORTC study): a phase III randomised trial. Lancet. 2002;360(9327):103-106. 10.1016/s0140-6736(02)09408-4 12126818

